# Science and Art of Obstetrics

**Published:** 1891-03

**Authors:** 


THE SCIENCE AND ART OF OBSTETRICS. By Theophilus Parvin, M. D., LL. D., Professor of obstetrics and diseases of women and children in Jefferson Medical College, Philadelphia. Second edition revised and enlarged: Lea Bros & Co.

The above is a valuable contribution to the practice of modern midwifery, easily ranking with those other classics on the obstetric art, the works of Playfair and Leishman. There is probably no one better qualified to write and speak on this subject than Dr. Parvin, and he has done so in a masterly way. While not departing too much from reliable landmarks the author has brought his book thoroughly abreast with the time. In this regard we refer specifically to the sections of Ectopic Pregnancy, and antisepsis in Labor.

If we were to find fault at all it would be with the arrangement of subjects. But, on the whole, excellent.

